# Associação Dose-Resposta entre Trajetórias de Intensidade da Atividade Física no Lazer e Diabetes entre Homens e Mulheres no ELSA-Brasil

**DOI:** 10.36660/abc.20250091

**Published:** 2025-08-12

**Authors:** André Luis Messias dos Santos Duque, Daniela Polessa Paula, Danilo de Paula Santos, Maria Del Carmen Bisi Molina, Luana Giatii, Maria Inês Schmidt, Maria de Jesus Mendes da Fonseca, Rosane Harter Griep

**Affiliations:** 1 Prefeitura Municipal de Petrópolis Secretaria de Educação Petrópolis RJ Brasil Prefeitura Municipal de Petrópolis - Secretaria de Educação, Petrópolis, RJ – Brasil; 2 Escola Nacional de Ciências Estatísticas Rio de Janeiro RJ Brasil Escola Nacional de Ciências Estatísticas, Rio de Janeiro, RJ – Brasil; 3 Universidade Federal do Rio Grande do Sul Porto Alegre RS Brasil Universidade Federal do Rio Grande do Sul, Porto Alegre, RS – Brasil; 4 Universidade Federal do Espírito Santo Vitória ES Brasil Universidade Federal do Espírito Santo, Vitória, ES – Brasil; 5 Universidade Federal de Minas Gerais Belo Horizonte MG Brasil Universidade Federal de Minas Gerais, Belo Horizonte, MG – Brasil; 6 Universidade Federal do Rio Grande do Sul Porto Alegre RS Brasil Universidade Federal do Rio Grande do Sul, Porto Alegre, RS – Brasil; 7 Fundação Oswaldo Cruz Rio de Janeiro RJ Brasil Fundação Oswaldo Cruz (Fiocruz), Rio de Janeiro, RJ – Brasil; 8 Escola Nacional de Saúde Pública Rio de Janeiro RJ Brasil Escola Nacional de Saúde Pública, Rio de Janeiro, RJ – Brasil

**Keywords:** Exercício Físico, Trajetória de Vida, Diabetes Mellitus Tipo 2, Estilo de Vida

## Abstract

**Fundamento:**

A atividade física (AF) desempenha um papel fundamental na prevenção do Diabetes Mellitus tipo 2 (DM-2). No entanto, os achados sobre a influência da intensidade da AF no DM-2 ao longo do tempo ainda são inconsistentes.

**Objetivo:**

Examinar a associação dose-resposta entre trajetórias de intensidade da AF no lazer e DM-2.

**Métodos:**

O estudo incluiu dados basais do Estudo Longitudinal de Saúde do Adulto (ELSA-Brasil) (2008-2010) e ao longo de 11 anos de acompanhamento de 5.777 mulheres e 4.590 homens, com idades entre 35 e 75 anos. As trajetórias de intensidade da AF no lazer foram avaliadas por meio do Questionário Internacional de Atividade Física (IPAQ), enquanto o DM-2 foi identificado por autorrelato, uso de medicação ou critérios laboratoriais. Foi utilizada regressão logística ordinal para estimar razões de chances (OR) e intervalos de confiança de 95% (IC95%).

**Resultados:**

Uma menor proporção de participantes com DM-2 (14,4% dos homens e 5% das mulheres) e uma maior proporção sem diabetes (22,1% dos homens e 40,8% das mulheres) foram observadas entre aqueles com trajetória de alta intensidade. Comparado às trajetórias de intensidade moderada, a alta intensidade conferiu proteção contra DM-2: OR 0,63 (IC95% = 0,40-0,98) para homens, e OR 0,33 (IC95% = 0,14-0,79) para mulheres, e as trajetórias de baixa intensidade conferiu maior chance de pré-diabetes entre os homens [OR = 1,36 (IC95% = 1,09-1,69)].

**Conclusão:**

A AF de maior intensidade ao longo do tempo esteve associada a uma menor proporção de casos de DM-2 em homens e mulheres. Assim, programas visando a prevenção e o controle do DM-2 devem enfatizar a importância da manutenção de atividades de alta intensidade ao longo do tempo.

## Graphical abstract

**Figura Central:**
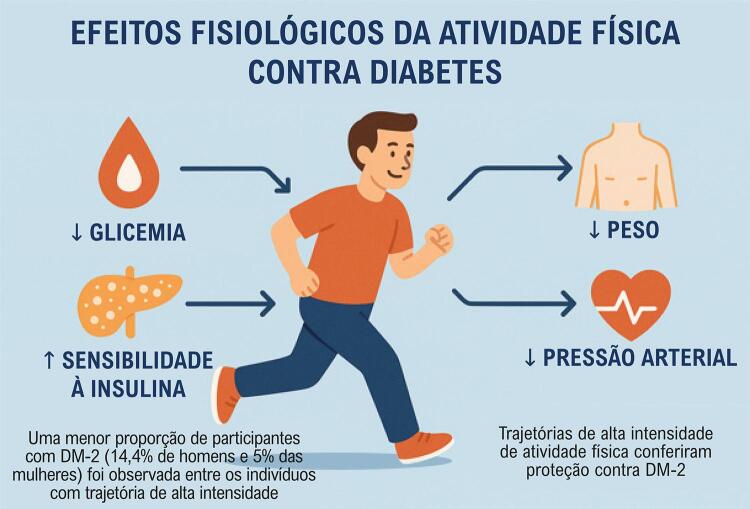
Associação Dose-Resposta entre Trajetórias de Intensidade da Atividade Física no Lazer e Diabetes entre Homens e Mulheres no ELSA-Brasil

Alguns dos efeitos fisiológicos da atividade física que conferem proteção contra o diabetes.

## Introdução

Diabetes mellitus (DM) é um grupo de distúrbios do metabolismo dos carboidratos que ocorre quando a glicose é subutilizada como fonte de energia ou superproduzida devido à glicogênese e glicogenólise prejudicadas, resultando em hiperglicemia.^1^O DM é um importante problema de saúde pública: segundo a *International Diabetes Federation* (IDF), 537 milhões de pessoas tinham diabetes em 2021, número que pode subir para 643 milhões em 2030 e 783 milhões até 2045. No Brasil, a doença afeta 12,5 milhões de pessoas, e esse número deve chegar a 20,3 milhões em 2045.^2 , 3^

O DM é um fator de risco relevante para doenças cardiovasculares, cegueira, insuficiência renal e amputação de membros inferiores. Entre 2000 e 2019, a taxa de mortalidade por DM aumentou 13% em países de baixa e média renda.^4^Como resultado, o DM está associado a altos custos de saúde: os gastos relacionados ao DM foram de aproximadamente US$ 43 bilhões no Brasil em 2021, tornando o país o terceiro com os maiores custos relacionados ao DM no mundo.^2^

O DM tipo 2 (DM-2), que representa mais de 95% dos casos, resulta de uma interação complexa entre genética e estilo de vida.^5^ Medidas relacionadas ao estilo de vida, como redução do peso corporal, mudança nos hábitos alimentares e aumento da Atividade Física (AF), são essenciais para o combate à doença.^6^A importância da AF no enfrentamento do DM-2 tem sido amplamente destacada na literatura,^7 - 9^ e a AF deve ser promovida como uma estratégia prioritária para a prevenção da doença.^10^

Os benefícios da AF associados à prevenção do DM-2 incluem redução da resistência à insulina, aumento da secreção de insulina, melhora da função das células beta-pancreáticas e da proteína transportadora de glicose (GLUT-4), maior utilização da glicose para produção de energia, redução do tecido adiposo, e melhora da sensibilidade à insulina.^11^Devido aos benefícios fisiológicos resultantes da prática de AF ao longo do tempo, a literatura científica tem enfatizado cada vez mais sua importância no combate ao DM-2. A Figura Central apresenta alguns dos efeitos fisiológicos da AF que protegem contra o diabetes.

Em um estudo recente que analisou os padrões das trajetórias de AF na redução do risco de DM-2, foram examinados dados de 99 532 participantes recrutados no Reino Unido. Os resultados indicaram que trajetórias de AF de maior intensidade reduziram significativamente o risco de DM-2, destacando a importância de praticar AF ao longo do tempo, especialmente em intensidades mais altas.^7^

No entanto, o efeito dose-resposta entre intensidade da AF e risco de DM-2 ainda é inconsistente na literatura, e não está claro qual intensidade de AF proporciona os maiores benefícios no combate à doença. Em uma metanálise e revisão sistemática^12^ que investigou a associação entre tipos específicos de AF e o risco de DM-2, foram observados benefícios significativos para AF de intensidade leve, moderada ou vigorosa, com reduções mais pronunciadas no risco de DM-2 em níveis mais baixos de AF. Por outro lado, uma revisão sistemática e meta-análise de estudos de coorte prospectivos que analisou os resultados de pesquisas sobre a associação entre AF ao longo do tempo e a incidência de DM-2 concluiu que níveis mais altos de AF (maior duração ou intensidade) em momentos de lazer estavam associados a uma incidência significativamente menor de DM-2.^13^Em contraste, Koloverou et al.^14^ analisaram o efeito da AF na incidência de DM-2 ao longo de 10 anos e encontraram uma redução de 53% na incidência da doença para AF moderada, em comparação com AF leve, mas não identificaram resultados significativos para AF intensa.

Diante do exposto, torna-se evidente que novos estudos longitudinais são necessários para explorar a associação entre diferentes intensidades de AF ao longo do tempo e o DM-2, visando estratégias mais eficazes para enfrentar a doença. Assim, este estudo tem como objetivo analisar a relação dose-resposta entre trajetórias de intensidade da AF de lazer e DM-2, comparando os resultados entre homens e mulheres participantes de um estudo longitudinal brasileiro.

## Métodos

### Estudo ELSA-Brasil: população e amostra

Os dados foram extraídos do Estudo Longitudinal de Saúde do Adulto (ELSA-Brasil), um estudo prospectivo, multicêntrico, do tipo coorte, conduzido em cinco instituições de ensino superior e uma instituição de pesquisa nas regiões Nordeste, Sul e Sudeste do Brasil. O estudo investigou fatores relacionados ao desenvolvimento e à progressão de doenças crônicas não transmissíveis. No basal, (2008-2010), o ELSA-Brasil incluiu 15105 servidores públicos ativos e aposentados, com idades entre 35 e 74 anos, provenientes de cinco universidades (Universidade Federal da Bahia, Universidade Federal de Minas Gerais, Universidade Federal do Espírito Santo, Universidade Federal do Rio Grande do Sul e Universidade de São Paulo) e uma instituição de pesquisa (Fundação Oswaldo Cruz). Até o momento, foram realizadas três visitas presenciais de acompanhamento (2012-2014, 2017-2019 e 2022-2024). Técnicos treinados e certificados conduziram testes clínicos e antropométricos, além de entrevistas detalhadas por meio de questionários.^15 - 17^

O ELSA-Brasil foi aprovado pela Comissão Nacional de Ética em Pesquisa (CONEP) e por todos os comitês de ética em pesquisa dos seis centros de estudo envolvidos. Todos os participantes assinaram um termo de consentimento informado, garantindo a segurança e confidencialidade dos dados. Este estudo foi aprovado pelo comitê de ética em pesquisa da Escola Nacional de Saúde Pública (ENSP/Fiocruz) (CAEE: 61848922.7.0000.5240).

Os participantes dos seis centros de pesquisa foram considerados elegíveis se responderam aos questionários sobre AF no basal e nas duas primeiras visitas de acompanhamento, além de fornecerem informações sobre o desfecho. Foram excluídos os casos de óbitos ocorridos ao longo do tempo, assim como os participantes com dados de AF não plausíveis (> 840 minutos/semana de AF leve, > 630 minutos/semana de AF moderada ou > 420 minutos/semana de AF intensa).^18^

### Variável de exposição

A AF foi avaliada por meio da seção de AF no lazer do Questionário Internacional de Atividade Física (IPAQ – versão longa) em três momentos (basal e duas primeiras visitas de acompanhamento). Essa seção inclui questões sobre a frequência, intensidade e duração da AF no lazer, medidas em minutos por semana, obtidas pela multiplicação da duração de cada atividade realizada pela sua frequência semanal, e classificadas em atividades de intensidade leve, moderada e intensa.^19^ A intensidade da AF refere-se ao esforço físico necessário para realizar uma atividade e pode ser leve, moderada ou intensa. Está relacionada ao gasto energético e pode ser expressa em múltiplos de Equivalentes Metabólicos da Tarefa (METs), calculados com base na quantidade de AF realizada. O MET semanal é obtido pela multiplicação da frequência semanal pela duração da AF realizada, considerando 3,3 METs para AF leve, 4,0 METs para AF moderada e 8,0 METs para AF intensa.

A intensidade da AF foi classificada com base nos METs semanais dos participantes e categorizada, em cada um dos três momentos, como leve (participante relatou não praticar AF ou fazê-lo em menor intensidade do que nas outras categorias); moderada (participante atingiu 600 MET-min/semana), ou intensa (participante atingiu 1500 MET-min/semana em AF intensa ou 3000 MET-min/semana em uma combinação de AF leve, moderada ou intensa).^18^

As trajetórias de AF ao longo dos três momentos (2008-10, 2012-14 e 2017-19) foram especificadas como leve, moderada ou intensa quando essa intensidade ocorreu em dois ou mais momentos. Os participantes com três classificações de intensidade diferentes foram considerados como “sem padrão”.

### Desfecho

Para o diagnóstico de diabetes, são utilizados os critérios propostos pela IDF, que consideram alterações na glicemia de jejum e na glicemia após a ingestão de 75g de glicose oral.^2^ Além disso, a avaliação da hemoglobina glicada (HbA1c) – a fração de hemoglobina que se liga à glicose – também é utilizada nos critérios diagnósticos da doença.^2^O pré-diabetes, que se refere a pessoas com tolerância à glicose comprometida e/ou glicemia de jejum alterada, está associado a um risco aumentado de desenvolver DM-2 e complicações relacionadas, sendo também baseado nos critérios propostos pela IDF.^2^

A classificação do DM-2 foi baseada em exames laboratoriais, informações autorrelatadas sobre diagnóstico médico prévio e uso de medicação, coletadas na segunda visita de acompanhamento (2017-2019). Foram coletadas amostras de sangue por punção venosa após 12 horas de jejum, com medição da glicemia de jejum e HbA1c, seguida de um teste padronizado de tolerância à glicose com 75g e uma segunda amostra de sangue coletada duas horas depois para medir a glicemia sérica.^17^ Os participantes que relataram um diagnóstico prévio ou usaram medicação para diabetes nas últimas duas semanas foram classificados como diabéticos. Os participantes sem diagnóstico prévio de diabetes foram classificados como diabéticos se apresentassem glicemia de jejum ≥ 126 mg/dL, glicemia após duas horas ≥ 200 mg/dL, ou HbA1c ≥ 6,5%. Os participantes foram classificados como pré-diabéticos se apresentassem glicemia de jejum entre 100 mg/dL e 125 mg/dL, glicemia após duas horas entre 140 mg/dL e 200 mg/dL, HbA1c entre 5,8% e 6,4%.^20^

### Covariáveis

Foram selecionadas covariáveis sociodemográficas, comportamentais e clínicas associadas à AF e ao DM-2, com base na literatura,^21 - 23^ que pudessem ser usadas para ajuste dos modelos. Essas variáveis incluíram sexo (masculino/feminino), idade (35-44, 45-54, 55-64 e ≥ 65 anos), renda per capita, raça/cor autorrelatada (branca ou não branca; esta última incluindo preta, parda, amarela e indígena), estado civil (com parceiro ou sem parceiro; este último incluindo divorciado, solteiro ou viúvo), situação de emprego (ativo ou aposentado), escolaridade – alta, ou seja, ensino superior; ou baixa escolaridade (ensino médio completo ou menos), abuso de álcool (Sim/Não, definido como ≥ 140 gramas de álcool por semana para mulheres e ≥ 210 para homens), tabagismo (ex-fumante, fumante ou nunca fumou), consumo diário de frutas (Sim/Não), consumo diário de vegetais (Sim/Não). As variáveis de condição de saúde incluíram hipertrigliceridemia (Sim/Não, definida como triglicerídeos ≥ 150 mg/dL), estado nutricional, e hipertensão arterial. O estado nutricional foi obtido por meio do Índice de Massa Corporal (IMC), calculado pela divisão do peso (balança eletrônica Toledo^®^) pela altura ao quadrado. Os participantes foram classificados como não obesos (IMC < 30kg/m^2^) ou obesos (IMC ≥ 30kg/m^2^). A hipertensão arterial (Sim/Não) foi definida como pressão arterial ≥ 140/90 mmHg ou uso de medicação anti-hipertensiva. A pressão arterial casual foi medida no braço esquerdo, após cinco minutos de repouso, utilizando um monitor de pressão oscilométrico validado (Omron HEM 705CPINT, EUA), com o participante sentado em um ambiente tranquilo e temperatura controlada (20-24°C). Foram realizadas três medições em intervalos de um minuto, e a pressão arterial casual foi calculada como a média das duas últimas medições.

### Análise dos dados

Todas as análises foram estratificadas por sexo e realizadas utilizando o software R, versão 4.2.2.^24^ A análise descritiva das variáveis sociodemográficas, clínicas e comportamentais utilizou frequências absolutas e relativas. As proporções foram comparadas por meio do teste qui-quadrado para variáveis categóricas e do teste t de *Student* pareado para variáveis contínuas, considerando um nível de significância de 5%. A variável renda (contínua) foi descrita utilizando média ± desvio padrão.

Foram estimados modelos de regressão logística ordinal, com os resultados expressos em razões de chances (ORs) e respectivos intervalos de confiança de 95% (IC95%), para avaliar a associação entre trajetória da intensidade da AF e DM-2. Com base na literatura, os efeitos específicos das trajetórias de intensidade da AF sobre o DM-2 foram avaliados por meio da seleção de variáveis de ajuste sociodemográficas (idade, renda, escolaridade, raça/cor, estado civil) que deveriam estar associadas à exposição e ao desfecho, além de precederem o desfecho. A modelagem começou com o modelo completo, e as variáveis de ajuste estatisticamente não significativas foram removidas uma a uma. Os modelos de ajuste foram avaliados utilizando o Critério de Informação de Akaike (AIC), a categoria de referência foi AF em intensidade moderada.

## Resultados

**Figura 1:**
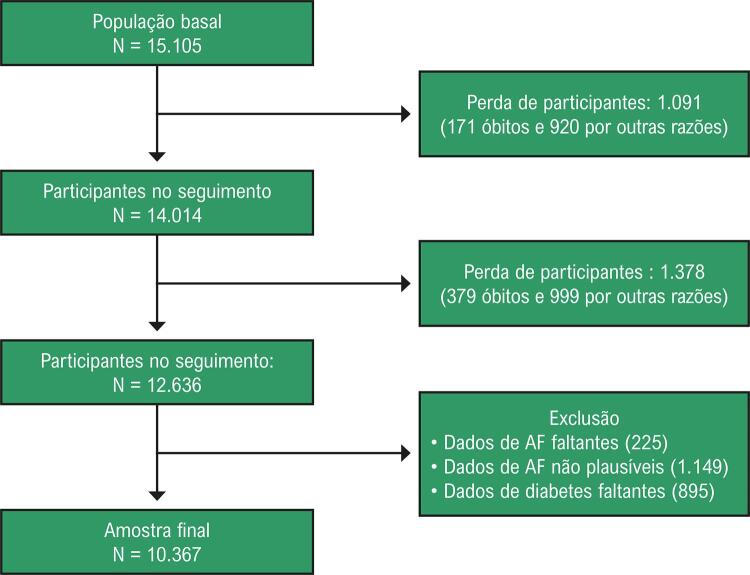
– Critérios de seleção da amostra do estudo, ELSA-Brasil (2008-2019).

Dos 15 105 participantes do momento basal do ELSA-Brasil, 12 636 atenderam aos critérios de inclusão (fornecendo dados de AF no basal e nas duas visitas de acompanhamento). Após o cálculo das perdas de seguimento, valores não plausíveis e dados falantes de AF ou DM-2, a amostra final foi composta por 10 367 participantes, dos quais 5777 (55,7%) eram mulheres e 4590 (44,3%) eram homens ( Figura 1 ).

A proporção total de participantes com DM-2 foi de 20% na segunda visita de seguimento (23,2% dos homens e 18% das mulheres). A Tabela 1 apresenta uma descrição dos participantes com base em variáveis sociodemográficas, comportamentais e clínicas, de acordo com a classificação do diabetes. De modo geral, o DM-2 aumentou com a idade e foi mais frequente entre participantes com uma trajetória de baixa intensidade da AF, níveis mais baixos de escolaridade, raça/etnia autorrelatada como não branca, aposentados, ex-fumantes, aqueles que não relataram consumo abusivo de álcool, aqueles que consumiam frutas diariamente e aqueles classificados com hipertrigliceridemia e hipertensão. A proporção de DM-2 diminuiu significativamente quando as trajetórias de intensidade da AF passaram de moderada para intensa, especialmente entre mulheres.

**Tabela 1:** – Descrição da população do estudo pelas variáveis sociodemográficas, comportamentais e clínicas basais, sexo e classificação do diabetes na segunda visita de acompanhamento; Estudo Longitudinal de Saúde do Adulto (ELSA-Brasil), 2008-2019

Características	Homens (N = 4590)			Mulheres (N = 5777)		
	Normal	Pré-diabéticos	Diabéticos	Normal	Pré-diabéticos	Diabéticos
	n = 797 (17,3%)	n = 2371 (59,5%)	n = 1062 (23,2%)	n = 1522 (26,3%)	n = 3214 (55,6%)	n = 1041 (18,1%)
Trajetória da intensidade da atividade física *						
Fraca	16,6	59,3	24,1	25,5	55,8	18,7
Moderada	20,1	57,5	22,3	28,5	54,2	17,3
Alta	22,1	63,5	14,4	40,8	54,2	5,0
Sem padrão	14,8	65,9	19,3	32,1	57,2	10,7
Grupo etário (anos)*						
35 - 44	27,5	60,5	11,9	42,0	48,6	9,4
45 - 54	16,2	60,0	23,7	24,8	58,3	16,9
55 - 64	11,8	58,7	29,5	20,0	57,0	23,0
≥ 65	11,1	56,5	32,5	12,4	56,4	31,2
Escolaridade*						
Alta	20,0	60,3	19,7	31,2	54,5	14,3
Baixa	14,5	58,6	26,9	20,4	57,0	22,6
Renda per capita (média e desvio padrão)***	3.646,12 ± 2.625,29	2.625,29 ± 2.991,99	3.567,55 ± 3.050,03	4.229,28 ± 3.235,25	3.984,41 ± 3.169,99	3.563,11 ± 3.142,55
Raça/cor da pele autorrelatada *						
Não branco	34,1	12,2	53,7	22,2	56,0	21,8
Branco	21,1	60,5	21,1	30,1	55,3	14,6
Estado civil***						
Sem parceiro	19,0	60,2	20,8	24,5	55,3	20,2
Com parceiro	17,0	59,4	23,6	27,9	55,9	16,2
Situação de emprego n*						
Ativo	18,5	60,0	21,5	29,1	55,4	15,5
Aposentado	10,3	56,6	33,1	15,9	56,7	27,4
Tabagismo*						
Nunca fumou	20,1	60,5	19,3	28,3	55,0	16,6
Ex-fumante	13,8	57,5	28,7	22,6	56,0	21,4
Fumante atual	15,9	61,3	22,8	23,4	58,1	18,4
**Abuso de álcool**						
Não	17,8	58,9	23,3	26,6	55,4	18,1
Sim	13,9	64,1	22,0	20,1	64,4	15,5
Consumo diário de frutas ***						
Não	17,3	61,0	21,7	29,0	55,9	15,1
Sim	17,4	57,9	24,7	24,9	55,5	19,6
Consumo diário de vegetais***						
Não	16,8	60,8	22,4	27,3	54,8	17,9
Sim	18,0	58,0	24,0	25,7	56,2	18,1
Estado nutricional**						
Não obeso	20,5	61,0	18,4	31,9	55,5	12,6
Obeso	7,0	54,5	38,4	13,0	55,9	31,1
Hipertrigliceridemia*						
Não	21,1	60,5	18,4	30,1	55,6	14,3
Sim	11,1	57,9	31,0	12,0	55,6	32,4
Hipertensão arterial*						
Não	23,6	63,0	13,4	33,5	56,1	10,3
Sim	9,6	55,1	35,3	14,4	54,8	30,8

*p<0,05 para ambos os sexos; **p<0,05 para homens; ***p<0,05 para mulheres.

**Figura 2:**
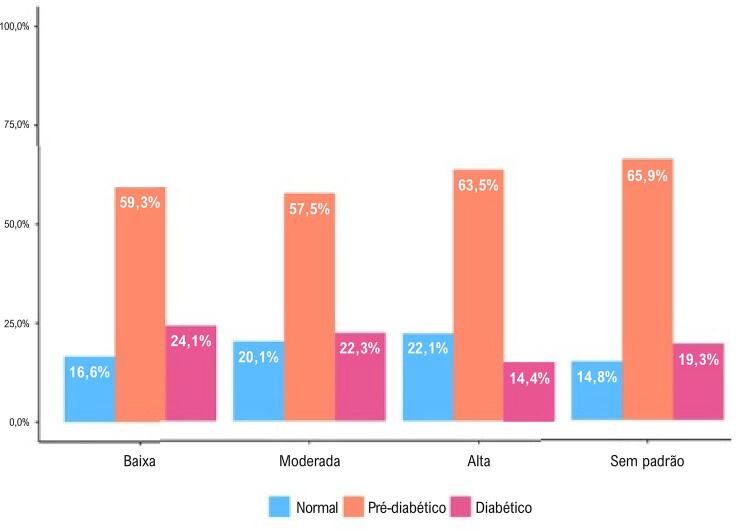
– Classificação dos homens por trajetória de intensidade da atividade física; Estudo Longitudinal de Saúde do Adulto (ELSA-Brasil), 2008-2019.

Nas Figuras 2 e 3 , é possível observar a relação dose-resposta entre as trajetórias da intensidade da AF e a classificação dos participantes. Em ambos os sexos, maior intensidade de AF ao longo do tempo foi associada a menores proporções de DM-2. Entre os homens, as proporções foram 24,1%, 22,3% e 14,4%, respectivamente, para as trajetórias de AF leve, moderada e intensa. Entre as mulheres, as proporções foram 18,7%, 17,3% e 5%, respectivamente. Para os homens e mulheres classificados como “sem padrão” de trajetória da intensidade de AF, as proporções foram 19,3% e 10,7%, respectivamente ( Figuras 2 e 3 ).

**Figura 3:**
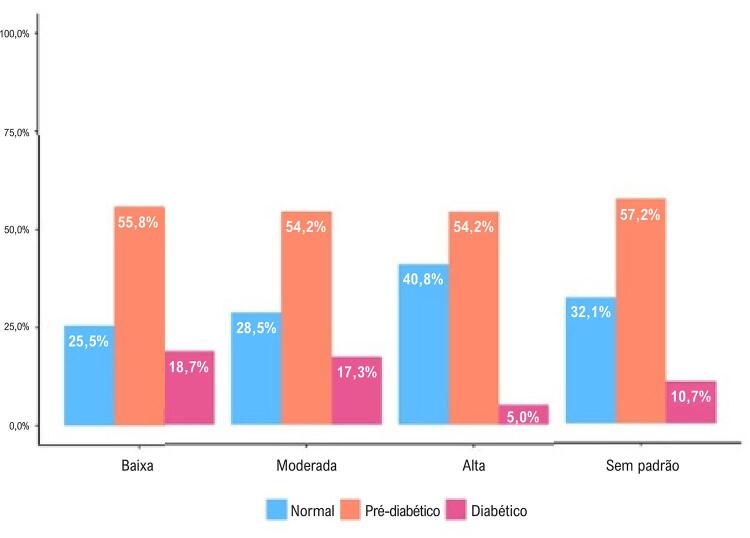
– Classificação das mulheres por trajetória de intensidade da atividade física; Estudo Longitudinal de Saúde do Adulto (ELSA-Brasil), 2008-2019.

As associações ajustadas para variáveis sociodemográficas mostraram que, em comparação com a trajetória moderada, a intensidade forte proporcionou proteção contra DM-2 em relação ao pré-diabetes [OR = 0,63 (IC95% = 0,40-0,98) para homens, OR = 0,33 (IC95% = 0,14-0,79) para mulheres]. Por outro lado, entre os homens classificados como normais, a trajetória de AF leve foi associada a maior chance de o participante ser pré-diabético [OR = 1,36 (IC95% = 1,09-1,69)] ( Tabela 2 ). Para os homens, a associação entre trajetórias de intensidade da AF e DM-2 foi mais bem analisada pelo modelo ajustado por idade, escolaridade, raça/cor, ocupação e renda *per capita* . Para as mulheres, o melhor modelo foi o ajustado por idade, escolaridade, raça/cor, ocupação e renda per capita.

**Tabela 2:** – Descrição da população do estudo com base em variáveis sociodemográficas, comportamentais e clínicas da linha de base, sexo e classificação do diabetes na segunda visita de acompanhamento; Estudo Longitudinal de Saúde do Adulto (ELSA-Brasil), 2008-2019

Classificação	Trajetórias da intensidade da atividade física	Homens		Mulheres	
		OR bruto (IC95%)	OR ajustado *(IC95%)	OR bruto (IC95%)	OR ajustado **(IC95%)
Pré-diabetes/ Normal	Alta	0,99 (0,68; 1,44)	1,18 (0,81; 1,74)	0,71 (0,47; 1,07)	0,83 (0,54; 1,26)
	Baixa	1,24 (1,00; 1,54)	1,36 (1,09; 1,69)	1,17 (0,97; 1,41)	1,20 (0,99; 1,45)
Diabetes/ Pré-diabetes	Alta	0,53 (0,34; 0,83)	0,63 (0,40; 0,98)	0,28 (0,12; 0,68)	0,33 (0,14; 0,79)
	Baixa	1,04 (0,85; 1,27)	1,10 (0,89; 1,35)	1,03 (0,83; 1,27)	1,03 (0,82; 1,28)

* ajustado por grupo etário, escolaridade e raça/cor autorrelatada. ** ajustado por grupo etário, escolaridade, raça/cor autorrelatada, situação de emprego e renda per capita.

## Discussão

Em nosso conhecimento, este é o primeiro estudo longitudinal brasileiro a examinar a associação dose-resposta entre trajetórias de intensidade da AF e DM-2, além de variáveis sociodemográficas, comportamentais e clínicas, em três momentos distintos.

Os resultados indicam que, para ambos os sexos, uma trajetória de alta intensidade da AF esteve associada a menores chances de o participante ser classificado como portador de DM-2. A AF de alta intensidade tem sido identificada como essencial para promover a saúde e prevenir doenças.^25 - 28^Por exemplo, uma revisão recente, que considerou diferentes delineamentos de estudo, indicou que a AF de alta intensidade confere maiores benefícios à saúde, reduz o risco de doenças crônicas não transmissíveis e melhora a saúde mental, sugerindo que novas recomendações para a prática de AF deveriam enfatizar atividade de alta intensidade.^25^

A AF de alta intensidade é uma maneira eficiente de promover a saúde sem exigir muito tempo.^26 , 27^A literatura científica apresenta descobertas interessantes que motivam a prática de AF de alta intensidade. Um ensaio clínico randomizado investigou o efeito do treinamento intervalado de alta intensidade e baixo volume sobre o risco cardiometabólico e a capacidade de exercício em mulheres com DM-2 e idade média de 44,5 anos. Após 16 semanas de acompanhamento, foram observados benefícios significativos na glicemia de jejum, na HbA1c, na pressão arterial sistólica, nos níveis de colesterol HDL e triglicerídeos, no peso corporal e IMC, na circunferência da cintura, e na gordura subcutânea. Além disso, concluiu-se que as mulheres que participaram do programa de AF apresentaram redução nas doses diárias de medicamentos hipoglicemiantes e anti-hipertensivos, e que o tempo semanal necessário para alcançar esses benefícios foi 25 a 56% menor do que o recomendado.^29^

Mais recentemente, um estudo realizado com dados de 71 893 adultos no Reino Unido, com um tempo médio de acompanhamento de 5,9 anos, encontrou uma associação dose-resposta entre AF intensa e redução da mortalidade (por todas as causas, doenças cardiovasculares e câncer): risco absoluto de 2,12% para > 0 e < 10 minutos de AF intensa; 1,78% para 10 a < 30 minutos; 1,47% para 30 a < 60 minutos; 1,10% para > 60 minutos. Os autores concluíram que 15 a 20 minutos por semana de AF intensa estavam associados a uma redução de 16 a 40% na taxa de mortalidade (todas as causas, doenças cardiovasculares e câncer), com reduções adicionais ocorrendo até 50 a 57 minutos por semana.^26^Outro estudo,^27^ com dados de 25 241 participantes, com idade média de 61,8 anos e 6,9 anos de acompanhamento, concluiu que apenas 4,4 minutos por dia de AF intensa reduziram o risco de mortalidade por todas as causas e por câncer em 26 a 30%, além de reduzir o risco de mortalidade por doenças cardiovasculares em 32 a 34%.^27^

Um estudo longitudinal recente, com um tempo médio de acompanhamento de 6,8 anos, examinou dados de 70 830 participantes britânicos, com idade média de 61,6 anos, e investigou associações entre AF e obesidade abdominal com o risco de doenças cardiovasculares.^28^Os autores concluíram que tanto a AF moderada quanto a AF intensa reduzem o risco de doenças cardiovasculares causadas pela obesidade abdominal, embora seja necessário cerca de 15 vezes mais AF moderada para alcançar resultados semelhantes aos obtidos com AF intensa.^28^

O estudo encontrou uma associação dose-resposta, em ambos os sexos, entre as trajetórias de intensidade da AF e o DM-2. Uma maior intensidade de AF esteve associada a uma redução progressiva na proporção de participantes com diabetes e a um aumento naqueles classificados como normais, corroborando uma revisão sistemática e metanálise dose-resposta.^12^ Após a análise de dados de 81 estudos, concluiu-se que a AF de alta intensidade esteve associada a uma maior redução no risco de DM-2 [RR = 0,61 (IC95% = 0,51-0,74)] do que a AF de intensidade moderada [RR = 0,68 (IC95% = 0,52-0,90)]. Outra revisão sistemática e metanálise, baseada em dados de 28 estudos longitudinais, que examinou associações dose-resposta entre AF no lazer e incidência de DM-2, também encontrou que o aumento da intensidade da AF gerou maiores benefícios.^13^

Reforçando esses achados, um estudo prospectivo do tipo coorte examinou as relações dose-resposta entre atividade física (AF) total/específica por intensidade e a incidência de diabetes mellitus tipo 2 (DM-2), considerando e estratificando por diferentes níveis de risco genético. Ao analisar dados de 59.325 participantes, com idade média de 61,1 anos e um tempo médio de acompanhamento de 6,8 anos, os autores encontraram uma associação linear entre AF de intensidade moderada e intensa e a incidência de DM-2, sugerindo que pessoas com alto risco genético de DM-2 devem praticar AF de intensidade moderada a intensa.^10^ No entanto, vale destacar que o estudo não separou AF moderada de intensa, tornando impossível identificar exatamente o papel da intensidade na proteção contra DM-2.

Da mesma forma, um estudo prospectivo^30^ realizado com dados do Reino Unido investigou a associação entre o ritmo de caminhada autorrelatado e a incidência de DM-2, explorando se o risco variaria com os níveis de AF e o tempo de caminhada. Após a análise de 4442 participantes, acompanhados por 7,4 anos, concluiu-se que caminhar em intensidade baixa ou moderada estava associado a um maior risco de DM-2, quando comparado à caminhada em alta intensidade.

Mais recentemente, um estudo examinou dados de 90044 participantes, com idade média de 56 anos (40 a 69 anos), para investigar como o volume (duração) e a intensidade de AF estavam associados à incidência de DM-2. Os autores concluíram que, para o mesmo volume de AF, a alta intensidade preveniu o DM-2 de forma mais eficaz do que a intensidade moderada ou leve [OR 0,88 (IC95% 0,85-0,91); OR 0,97 (IC95% 0,96-0,98); OR 0,99 (IC95% 0,98-1,00), respectivamente].^22^

Os efeitos preventivos da AF de alta intensidade podem ser explicados pelos mecanismos fisiológicos que ocorrem no organismo durante a prática, incluindo o aumento da captação de oxigênio. Durante a AF, a captação de oxigênio pode aumentar até 20 vezes, com incrementos ainda maiores nos músculos ativos. Essas demandas energéticas levam os músculos esqueléticos a esgotarem suas reservas de glicogênio, triglicerídeos e ácidos graxos.^11^Considerando que quanto mais intensa a AF, maior a necessidade energética, pode-se dizer que a AF intensa proporciona maiores efeitos protetores ao organismo. Além disso, a prática de AF em alta intensidade estimula o metabolismo anaeróbico alático e lático, promovendo um aumento nos níveis de GLUT-4, o que, por sua vez, melhora a captação periférica de glicose.^31^

Embora o DM-2 resulte de uma interação complexa que envolve elementos genéticos, socioeconômicos e comportamentais, há evidências científicas crescentes destacando a relevância da AF no manejo do DM-2, mesmo na presença de outros fatores de risco. Recentemente, um estudo investigou a associação entre duração do sono e diferentes intensidades de AF com o risco de DM-2 em estudo coorte populacional e, após a análise de dados de 88000 participantes, com idade média de 62,2 anos, foi constatado que um nível mais alto de AF, independentemente da intensidade, mitiga o risco de DM-2 causado pela curta duração do sono.^32^

Os pontos fortes do estudo incluem sua natureza inovadora, o diagnóstico de DM-2 por meio de testes clínicos e a avaliação da AF e de variáveis sociodemográficas, comportamentais e clínicas em três momentos distintos, além da separação das diferentes intensidades de AF e da estratificação por sexo.

No entanto, o estudo também apresenta limitações. Essas incluem o uso do gasto energético na AF para classificar a intensidade, uma medida que varia conforme o tipo de AF (corrida, natação, dança, ciclismo, musculação etc.), características individuais (sexo, composição corporal, peso, idade, biomecânica, nível de hidratação) e condições ambientais (temperatura, umidade). O IPAQ não identifica esses fatores e não há consenso na literatura sobre os valores a serem multiplicados para AF leve, moderada ou intensa. Porém, o IPAQ é um instrumento validado e amplamente utilizado em estudos populacionais. Além disso, embora existam outras formas de avaliar a prática de AF, como o uso de acelerômetros e tecnologias vestíveis, esses recursos são caros, o que dificulta sua aplicação em estudos com grandes populações.

Outra possível limitação do estudo é que o tipo de AF praticada pelos participantes não foi identificado e, assim, não foi possível determinar qual tipo de AF proporcionaria maiores benefícios no combate ao DM-2. No entanto, a literatura científica indica que tanto o exercício aeróbio como o treinamento de força conferem a mesma magnitude de proteção.^11^ Outra limitação deste estudo é o viés de sobrevivência, pois as trajetórias de intensidade da AF consideram três momentos distintos, enquanto os óbitos e perdas por DM-2 foram excluídos. Por fim, também é impossível estimar a direção de qualquer causalidade. Uma vez que os participantes foram classificados quanto ao DM-2 (normal, pré-diabético ou diabético) apenas na segunda visita de acompanhamento, não foi possível determinar se eles passaram a praticar AF de maior intensidade ao longo do tempo em resposta ao diagnóstico ou não. No entanto, foi observado que, ao longo dos anos, os participantes com trajetórias de AF de maior intensidade estavam mais protegidos contra o DM-2.

## Conclusão

Os resultados destacam a relevância da prática de AF de alta intensidade ao longo do tempo na prevenção do DM-2, sugerindo seu potencial como uma ferramenta não farmacológica essencial no combate à doença. Como os benefícios podem ser alcançados sem necessidade de longos períodos de atividade física, espera-se que os achados deste estudo contribuam para a melhoria das diretrizes de prevenção do DM-2 e forneçam um guia prático para a adoção da AF de alta intensidade de forma acessível à população.
