# Supplementary material for: Associação Dose-Resposta entre Trajetórias de Intensidade da Atividade Física no Lazer e Diabetes entre Homens e Mulheres no ELSA-Brasil
Source: Arq Bras Cardiol. 2025 Aug 12;122(8):e20250091. [Article in Portuguese] doi: 10.36660/abc.20250091 (PMC12677733; doi:10.36660/abc.20250091)
Supplement: Translation

# Translation: Dose-Response Association between Leisure-Time Physical Activity Intensity Trajectories and Diabetes among Men and Women in ELSA-Brasil

**DOI:** 10.36660/abc.20250091i

**Authors:** André Luis Messias dos Santos Duque, Daniela Polessa Paula, Danilo de Paula Santos, Maria Del Carmen Bisi Molina, Luana Giatii, Maria Inês Schmidt, Maria de Jesus Mendes da Fonseca, Rosane Harter Griep

**Affiliations:** 1 Brazil Prefeitura Municipal de Petrópolis - Secretaria de Educação, Petrópolis, RJ – Brazil; 2 Brazil Escola Nacional de Ciências Estatísticas, Rio de Janeiro, RJ – Brazil; 3 Brazil Universidade Federal do Rio Grande do Sul, Porto Alegre, RS – Brazil; 4 Brazil Universidade Federal do Espírito Santo, Vitória, ES – Brazil; 5 Brazil Universidade Federal de Minas Gerais, Belo Horizonte, MG – Brazil; 6 Brazil Universidade Federal do Rio Grande do Sul, Porto Alegre, RS – Brazil; 7 Brazil Fundação Oswaldo Cruz (Fiocruz), Rio de Janeiro, RJ – Brazil; 8 Brazil Escola Nacional de Saúde Pública, Rio de Janeiro, RJ – Brazil

**Keywords:** Physical Activity, Life Trajectory, Type 2 Diabetes Mellitus, Lifestyle

## Abstract

**Background:**

Physical activity (PA) plays a fundamental role in the prevention of type 2 diabetes mellitus (DM-2). However, findings regarding the influence of PA intensity on DM-2 over time remain inconsistent.

**Objective:**

To examine the dose-response association between leisure-time PA intensity trajectories and DM-2.

**Methods:**

The study included data from the Longitudinal Study of Adult Health (ELSA-Brasil), at baseline (2008-2010) and from 11 years’ follow-up, of 5777 women and 4590 men, aged from 35 to 75 years. Leisure-time PA intensity trajectories were assessed by means of the International Physical Activity Questionnaire, while DM-2 was identified by self-reporting, use of medication or laboratory criteria. Ordinal logistical regression was used to estimate odds ratios (OR) and 95% confidence intervals (95%CIs).

**Results:**

A lower proportion of participants with DM-2 (14.4% of men and 5% of women) and a higher proportion without diabetes (22.1% of men and 40.8% of women) were observed in those with a high-intensity trajectory. Compared to the moderate-intensity trajectory, high-intensity conferred protection against DM-2 (OR=0.63 [95% CI=0.40-0.98]) for men and women (OR=0.33 [95% CI=0.14-0.79]) and the low-intensity trajectory conferred a greater chance of pre-diabetes among men OR=1.36 [95% CI=1.09-1.69].

**Conclusion:**

Higher-intensity PA over time was associated with a lower proportion of DM-2 cases among men and women. Thus, programs aimed at preventing and controlling DM-2 should emphasize the importance of maintaining high-intensity activities over time.

## Introduction

Diabetes mellitus (DM) is a group of disorders of the carbohydrate metabolism that occurs when glucose is underutilised as an energy source or over-produced by impaired glycogenesis and glycogenolysis, resulting in hyperglycaemia.^1^DM is an important public health problem: the International Diabetes Federation (IDF) reported that 537 million people had diabetes in 2021, a figure that may rise to 643 million in 2030 and 783 million by 2045.^2^In Brazil, the disease affects 12.5 million people and this number is projected to reach 20.3 million in 2045.^3^

DM is an important risk factor for cardiovascular diseases, blindness, renal insufficiency and lower-limb amputation. Between 2000 and 2019 the DM mortality rate increased by 13% in low- and middle-income countries.^4^As a result, DM is associated with high health care costs: DM-related expenditure was approximately US$ 43 billion in Brazil in 2021, making it the country with the third-highest DM-related costs in the world.^2^

Type 2 DM (DM-2), which accounts for more than 95% of cases, results from a complex interaction between genetics and lifestyle.^5^Lifestyle measures, such as reducing body weight, changing eating habits and increasing physical activity (PA) are essential to combating the disease.^6^The importance of PA in combating DM-2 has been widely highlighted in the literature^7 - 9^and PA should be promoted as a priority strategy for preventing the disease.^10^

The benefits of PA that are associated with DM-2 prevention result from reduced insulin resistance, increased insulin secretion, improved beta-pancreatic cell function and production of glucose transporter protein (GLUT-4), greater use of glucose for energy production, reduction of adipose tissue, and improved insulin sensitivity.^11^Due to the physiological benefits resulting from the practice of PA over time, the scientific literature has increasingly emphasized its importance in tackling DM-2. The Central Illustration presents some of the physiological effects of PA that provide protection against diabetes.

In a recent study that analyzed patterns of PA trajectories for reducing the risk of DM-2, data from 99,532 participants recruited from the UK were examined. The results indicated that higher-intensity PA trajectories significantly reduced the risk of DM-2, highlighting the importance of engaging in PA over time, especially at higher intensities.^7^

However, the dose-response effect between PA intensity and the risk of DM-2 remains inconsistent in the literature, and it is unclear which PA intensity provides the greatest benefits in addressing the disease. In a meta-analysis and systematic review that investigated the association between specific types of PA and the risk of DM-2, significant benefits were observed for PA of light, moderate, or vigorous intensity, with more pronounced reductions in DM-2 risk at lower levels of PA.^12^In turn, a systematic review and meta-analysis of prospective cohort studies that analyzed the results of studies investigating the association between PA over time and the incidence of DM-2 concluded that higher levels of leisure-time PA (either greater duration or intensity) were associated with a significantly lower incidence of DM-2.^13^Conversely, Koloverou et al.^14^analyzed the effect of PA on the incidence of DM-2 over a 10-year period and found a 53% reduction in the incidence of the disease for moderate PA, when compared with low PA, and did not find significant results for strong PA.

Given the above, it becomes evident that new longitudinal studies are needed to explore the association between different intensities of PA over time and DM-2, aiming for more effective strategies to address the disease. Thus, this study aims to analyze the dose-response relationship between leisure-time PA intensity trajectories and DM-2, comparing the results between men and women participating in a Brazilian longitudinal study.

## Methods

### The ELSA-Brasil study: population and sample

Data were drawn from the Longitudinal Study of Adult Health ( *Estudo Longitudinal de Saúde do Adulto* , ELSA-Brasil), a prospective, multi-center cohort study conducted at five institutions of higher education and one research institution in Brazil’s northeast, south and southeast regions. The study investigated factors relating to the development and progression of non-communicable chronic diseases. At the baseline (2008-2010), ELSA-Brasil included 15,105 active and retired public servants, aged from 35 and 74 years, from five universities ( *Universidade Federal da Bahia* , *Universidade Federal de Minas Gerais* , *Universidade Federal do Espírito Santo* , *Universidade Federal do Rio Grande do Sul* and *Universidade de São Paulo* ) and one research institution ( *Fundação Oswaldo Cruz* ). To date, there have been three in-person follow-up visits (2012 to 2014, 2017 to 2019 and 2022 to 2024). Trained, certified research technicians conducted clinical and anthropomorphic tests and detailed interviews by questionnaire.^15 - 17^

ELSA-Brasil was approved by Brazil’s national research ethics committee ( *Comissão Nacional de Ética em Pesquisa* , CONEP) and by all the research ethics committees of the six study centers involved. All participants signed an informed consent form, and data secrecy and confidentiality were ensured. This study was approved by the research ethics committee of the *Escola Nacional de Saúde Pública* (ENSP/Fiocruz) (CAEE: 61848922.7.0000.5240).

Participants from the six research centers were considered eligible if they answered the questionnaires on PA at the baseline and at the first two follow-up visits and offered information on the outcome. Deaths occurring over time were excluded, as were participants with non-plausible PA data (> 840 minutes/week of weak PA, > 630 minutes/week of moderate PA or > 420 minutes/week of strong PA).^18^

### Exposure variable

PA was assessed by means of the leisure-time PA section of the International Physical Activity Questionnaire (IPAQ – long version) at three timepoints (baseline and first two follow-up visits). The section comprises questions on the frequency, intensity and duration of leisure-time PA, measured in minutes/week obtained by multiplying the duration of each of the activities performed by its weekly frequency, and classified into activities of weak, moderate and strong intensity. PA intensity is the degree of physical effort required to perform a PA and may be weak, moderate or strong.^19^It is related to energy expenditure and can be expressed in multiples of metabolic equivalents of task (METs), calculated by the amount of PA performed. Weekly MET is obtained by multiplying weekly frequency by the duration of PA performed, considering 3.3, 4.0 and 8.0 METs, respectively, to constitute weak, moderate and strong PA.

PA intensity was classified by participant’s weekly METs, and was categorized, at each of the three points in time, as either weak (participant reported not engaging in PA or doing so less than in the other categories); moderate (participant attained 600 MET-min/week) or strong (participant attained 1500 MET-min/week in strong PA or 3000 MET-min/week in a combination of weak, moderate or strong PA).^18^

The PA trajectory across the three points in time (2008-10, 2012-14 and 2017-19) was specified as of weak, moderate or strong intensity when that intensity occurred at two or more of the points in time. Participants with three different intensity classifications were considered “no pattern’’.

### Outcome

For the diagnosis of diabetes, the criteria proposed by the IDF are used, which consider changes in fasting blood glucose and blood glucose after ingestion of 75g of oral glucose.^2^In addition, the assessment of glycated hemoglobin (HbA1c) – the fraction of hemoglobin that binds to glucose – is also used in the diagnostic criteria for the disease.^2^Prediabetes, which refers to people with impaired glucose tolerance and/or fasting glucose and is associated with an increased risk of developing DM-2 and related complications, was also based on the criteria proposed by the IDF.^2^

DM-2 classification was based on laboratory tests, self-reported information of prior medical diagnosis and use of medication collected at the second follow-up visit (2017-2019). Blood samples were taken by 12-hour fasting venipuncture and measurement of fasting glycemia and HbA1c, followed by a standardized 75g glucose tolerance test and a second blood sample taken two hours later to measure serum glycemia.^17^Participants who reported a prior diagnosis or were using medication for diabetes in the past two weeks were classified as diabetic. Participants with no prior diagnosis of diabetes were classified as diabetic if their fasting glycaemia was ≥ 126 mg/dL; 2h glycaemia was ≥ 200 mg/dL or HbA1c ≥ 6.5%. Participants with fasting glycemia > 100mg/d and < 125 mg/dl; or 2-h glycaemia > 140 mg/dL and < 200 mg/d; or HbA1c ≥ 5.8 and < 6.4%,^20^were classified as prediabetic.

### Covariables

The sociodemographic, behavioral and clinical covariables were selected, based on the literature,^21 - 23^if they were associated with PA and DM-2 and could potentially be used to adjust the models. These were sex (male/female); age (35-44, 45-54, 55-64 and ≥ 65 years); per capita income; self-reported race/color (white or non-white; the latter comprising black, mixed, yellow and indigenous); marital status (partnered or not partnered, the latter comprising divorced, single or widowed); employment situation (active or retired); level of schooling (highly schooled, i.e., higher education, or little schooling, i.e., complete upper secondary education or less); alcohol abuse (Yes/No, defined as ≥ 140 grams of alcohol per week for women and ≥ 210 for men); tobacco use (former smoker, smoker or never smoked); ate fruit daily (Yes/No); and ate vegetables daily (Yes/No). The health condition variables were hypertriglyceridemia (Yes/No, defined as triglycerides ≥150 mg/dL); nutrition status; and arterial hypertension. Nutrition status was obtained by way of body mass index (BMI), calculated by dividing weight (Toledo® electronic scales) by height squared. Participants were classified as not obese (BMI < 30kg/m^2^) or obese (BMI ≥ 30kg/m^2^). Hypertension (Yes/No) was defined as arterial pressure of ≥ 140/90 mmHg or use of anti-hypertensive medication. Casual arterial pressure was measured on the left arm, after five minutes’ rest, using a validated oscillometric blood pressure monitor (Omron HEM 705CPINT, USA) with the participant seated in a quiet setting at controlled temperature (20-24°C). Three measurements were taken at one-minute intervals and casual PA was calculated as the mean of the last two of the three measurements.

Data were collected from the ELSA-Brasil baseline and two follow-up visits. Baseline data were used for the variables age, schooling, race/color, marital status and ate fruit and vegetables daily. For tobacco use, alcohol abuse, nutrition status and hypertriglyceridemia, the criterion was used in constructing the PA intensity trajectories (at least two identical responses were considered to classify the participant), so as to reflect consistent behavior over time. The information used for the variable “income” was from the second follow-up visit. For hypertension, participants who were diagnosed with the disease in at least one of the three moments were considered to have hypertension.

### Data analysis

All analyses were stratified by sex and performed using R software, version 4.2.2.^24^Descriptive analysis of the sociodemographic, clinical and behavioral variables used absolute and relative frequencies. Proportions were compared using the chi-square test for categorical variables and Student’s t-test paired for continuous variables, to a 5% level of significance. The income variable was described using mean ± standard deviation.

Ordinal logistic regression models were estimated, with results expressed in odds ratios (ORs) and respective 95% confidence intervals (95%CIs) to evaluate the association between PA intensity trajectories and DM-2. Drawing on the literature, the specific effects of PA intensity trajectories on DM-2 were assessed by selecting sociodemographic adjustment variables (age, income, schooling, race/color, marital status) that should be associated with exposure and outcome, as well as preceding the outcome. Modelling began with the complete model, and statistically non-significant adjustment variables were removed one by one. The adjustment models were evaluated using the Akaike information criterion (AIC) and the reference category was the moderate intensity trajectory.

## Results

Of the 15,105 baseline participants in ELSA-Brasil, 12,636 met the inclusion criteria (providing PA data at the baseline and the two follow-up visits). After computing losses to follow-up, non-plausible values and missing PA or DM-2 data, the final sample comprised 10,367 participants, 5,777 (55.7%) of whom were women and 4,590 (44.3%), men ( Figure 1 ).

The proportion of participants with DM-2 was 20% at the second follow-up visit (23.2% of the men and 18% of the women). Table 1 provides a description of participants based on sociodemographic, behavioral, and clinical variables by diabetes classification. In general, DM-2 increased with age and was more frequent among participants with a weak PA intensity trajectory, lower education levels, non-white self-reported race/ethnicity, retirees, former smokers, those who did not report abusive alcohol consumption, those who consumed fruits daily, and those classified with hypertriglyceridemia and hypertension. The proportion of DM-2 decreased significantly when the PA intensity trajectory shifted from moderate to strong, particularly among women.

In Figures 2 and 3 , it is possible to observe the dose-response relationship between the trajectories of PA intensity and the classification of participants. In both sexes, greater PA intensity over time was found to be associated with lower proportions of DM-2. Among the men were 24.1%, 22.3% and 14.4%, respectively, for the weak, moderate and strong PA trajectories; among women, the proportions were, respectively, 18.7%, 17.3% and 5%. For the men and women with no PA intensity trajectory pattern, the proportions were, respectively, 19.3% and 10.7% ( Figures 2 and 3 ).

Adjusted associations for sociodemographic variables showed that, compared to the moderate trajectory, strong intensity provided protection against type 2 diabetes in relation to pre-diabetes (OR = 0.63[95%CI = 0.40-0.98] for men and OR = 0.33[95%CI = 0.14-0.79] for women). Meanwhile, among the men classified as normal, the weak intensity trajectory was associated with greater odds of the participant’s being prediabetic (OR = 1.36, 95% CI = 1.09-1.69) ( Table 2 ). For the men, the association between PA intensity trajectories and DM-2 was best analyzed by the model adjusted by age, schooling and race/color. For the women, the best model was that adjusted by age, schooling, race/color, occupation and *per capita* income.

## Discussion

To our knowledge, this is the first longitudinal Brazilian study to examine dose-response association between PA intensity trajectories and DM-2 and related sociodemographic, behavioral and clinical variables at three points in time.

The results indicate that, for both sexes, a strong PA intensity trajectory was associated with smaller odds of the participant’s being classified as having DM-2. Strong intensity PA has been identified as essential to promoting health and preventing disease.^25 - 28^For example, a recent review that considered different study designs indicated that strong intensity PA conferred greater health benefits, reduced the risk of chronic noncommunicable diseases and improved mental health, and suggested that new recommendations for engaging in PA should emphasize strong intensity PA.^25^

Strong intensity PA is an efficient manner of promoting health without entailing spending major amounts of time.^26 , 27^The scientific literature offers interesting findings that motivate engagement in strong-intensity PA. A randomized clinical trial that investigated the effect of low-volume, high-intensity interval training on cardiometabolic risk and exercise capacity in women with DM-2 and mean age 44.5 years. At 16 weeks’ follow-up, they found significant benefits in fasting glycemia, HbA1c, systolic arterial pressure, HDL cholesterol, triglycerides, body weight, BMI, waist circumference and subcutaneous fat. They also concluded that women who took the PA program showed reductions in daily dosages of anti-hyperglycemic and anti-hypertensive medications and that the weekly time taken to achieve those benefits was 25 to 56% less than recommended.^29^

More recently, a study conducted with data from 71,893 adults in the United Kingdom, with mean follow up of 5.9 years, found a dose-response association between strong PA and reduced mortality (from all causes, cardiovascular diseases and cancer): absolute risk of 2.12% for > 0 and < 10 minutes of strong PA; 1.78% for 10 to < 30 minutes; 1.47% for 30 to < 60 minutes; 1.10% for > 60 minutes. They concluded that 15 to 20 minutes per week of strong PA was associated with a 16 to 40% lower mortality rate (all causes, cardiovascular diseases and cancer), with the additional reductions occurring up to 50 to 57 minutes per week.^26^In the same direction, a study^27^with data from 25,241 participants of mean age 61.8 years, with 6.9 years’ follow-up, concluded that only 4.4 minutes per day of strong PA reduced the risk of mortality from all causes and from cancer by 26 to 30%, in addition to reducing the risk of mortality from cardiovascular disease by 32 to 34%.^27^

A recent longitudinal study with a mean follow-up period of 6.8 years, which examined data from 70,830 British participants of mean age 61.6 years, investigated associations between PA and abdominal obesity with the risk of cardiovascular disease.^28^The authors concluded that both moderate and strong PA reduce the risk of cardiovascular disease caused by abdominal obesity, although about 15 times more moderate PA is required to achieve outcomes similar to those achieved with strong PA.^28^

The study found a dose-response association, in both sexes, between PA intensity trajectories and DM-2. Stronger PA intensity was associated with a progressive reduction in the proportion of participants with diabetes and an increase in those classified as normal, corroborating a systematic review and dose-response meta-analysis.^12^After analyzing data from 81 studies, it was concluded that strong-intensity PA was associated with a greater reduction in the risk of DM-2 (RRs 0.61 [95%CI 0.51-0.74]) than moderate-intensity PA (RR 0.68 [95%CI 0.52-0.90]). Another systematic review and meta-analysis of data from 28 longitudinal studies that examined for dose-response associations between leisure-time PA and incidence of DM-2, also found that increased PA intensity yielded greater benefits.^13^

Reinforcing these findings, a prospective cohort study examined the dose-response relationships between total/intensity-specific physical activity and incident DM-2 accounting for and stratified by different levels of genetic risk. Examining data from 59,325 participants with mean age 61.1 years and after a mean follow-up period of 6.8 years, the authors found a linear association between moderate and strong intensity PA and incidence of DM-2, suggesting that people at high genetic risk of DM-2 should engage in moderate to strong intensity PA.^10^Note, however, that they did not separate moderate from strong intensity, and thus it was not possible to identify exactly the role of intensity in protection against DM-2.

Similarly, a prospective study^30^using data from the United Kingdom investigated the association between self-reported walking pace and the incidence of DM-2, exploring whether the risk would differ according to PA levels and walking time. After analyzing 4,442 participants who were followed for 7.4 years, the authors concluded that walking at low or moderate intensity was associated with a higher risk for DM-2 when compared with walking at high intensity.

More recently, a study examined data from 90,044 participants with mean age 56 years (40 to 69 years), to investigate how PA volume (duration) and intensity were associated with incidence of DM-2. They concluded that, for the same PA volume, strong intensity prevented DM-2 more effectively than moderate or light intensity (OR 0.88 [95%CI 0.85-0.91]; OR 0.97 [95%CI 0.96-0.98]; OR 0.99 [95%CI 0.98-1.00]), respectively.^22^

The preventive effects of strong-intensity PA can be explained by the physiological mechanisms occurring in the organism during PA, which include increased oxygen intake. During PA, oxygen intake can increase by up to 20 times, with even greater increases in the active muscles. These energy needs lead skeletal muscles to deplete its reserves of glycogen, triglycerides and fatty acids.^11^Given that the more intense the PA the greater the energy needs, strong PA can be said to yield greater protective effects on the organism. Furthermore, practicing PA at a higher intensity stimulates anaerobic alactic and lactic metabolism, promoting an increase in GLUT-4 levels, which, in turn, enhances peripheral glucose uptake.^31^

Although DM-2 is the result of a complex interaction that encompasses genetic, socioeconomic, and behavioral elements, there is increasing scientific evidence highlighting the relevance of PA in DM-2 management, even in the presence of other risk factors. Recently, a study investigated the association of sleep duration and different intensities of PA with the risk of DM-2 in a population-based cohort study, and after analyzing data from 88,000 participants with a mean age of 62.2 years, it was found that a higher level of PA, regardless of intensity, mitigates the risk of DM-2 caused by short sleep duration.^32^

Strengths of the study include its innovative nature, the diagnosis of DM-2 by clinical tests and assessment of PA and sociodemographic, behavioral and clinical variables at three points in time, the separation of different intensities of PA and stratification by sex.

However, the study also has limitations. These include the use of energy expenditure in PA to classify the intensity, a measure that changes with PA type (running, swimming, dancing, cycling, weight training etc.), individual characteristics (sex, body composition, weight, age, biomechanics, level of hydration), and environmental conditions (of temperature, humidity). The IPAQ does not identify these factors and there is no consensus in the literature as to the values to be multiplied for weak, moderate or strong PA. All the same, the IPAQ is a validated instrument used in population studies. Furthermore, although there are other ways to assess the practice of PA, such as the use of accelerometers and wearable technology, these resources are expensive, which makes their use in studies with large populations difficult.

Another possible limitation of the study is that the type of PA participants engaged in was not identified, and it was not possible to identify what PA type would yield greatest benefits in combating DM-2. However, the literature does indicate that both aerobic exercise and strength training confer the same magnitude of protection.^11^Another limitation of this study is survival bias, because the PA intensity trajectories considered three points in time, while deaths and losses to DM-2 were excluded. Lastly, it is also impossible to estimate the direction of any causality, since participants were classified by DM-2 (normal, prediabetic or diabetic) only at the second follow-up visit, we could not determine whether participants engaged in greater intensity PA over time in response to their diagnosis or not. However, we did observe that, over the years, participants with greater-intensity PH trajectories were more protected against DM-2.

## Conclusion

The results highlight the relevance of engaging in high-intensity PA over time in the prevention of DM-2, suggesting its potential as an essential non-pharmacological tool in combating the disease. Since the benefits can be achieved without requiring long exercise duration, it is expected that the findings of this study will contribute to the improvement of DM-2 prevention guidelines and provide a practical guide for adopting high-intensity PA in a way that is accessible to the population.

**Figure 1:** – Study sample selection criteria, ELSA-Brasil (2008-2019).

**Figure 2:** – Classification of men by physical activity intensity trajectories, Longitudinal Study of Adult Health (ELSA-Brasil), 2008-2019.

**Figure 3:** – Classification of women by physical activity intensity trajectories, Longitudinal Study of Adult Health (ELSA-Brasil), 2008-2019.

**Table 1:** – Description of the study population by baseline sociodemographic, behavioral and clinical variables, sex, and diabetes classification at the second follow-up visit; Longitudinal Study of Adult Health (ELSA-Brasil), 2008-2019

Characteristics	Men (N = 4590)			Women (N = 5777)		
	Normal	Prediabetic	Diabetic	Normal	Prediabetic	Diabetic
	n = 797 (17.3%)	n = 2371 (59.5%)	n = 1062 (23.2%)	n = 1522 (26.3%)	n = 3214 (55.6%)	n = 1041 (18.1%)
Physical activity intensity trajectories*						
Weak	16.6	59.3	24.1	25.5	55.8	18.7
Moderate	20.1	57.5	22.3	28.5	54.2	17.3
Strong	22.1	63.5	14.4	40.8	54.2	5.0
No pattern	14.8	65.9	19.3	32.1	57.2	10.7
Age group (years)*						
35 to 44	27.5	60.5	11.9	42.0	48.6	9.4
45 to 54	16.2	60.0	23.7	24.8	58.3	16.9
55 to 64	11.8	58.7	29.5	20.0	57.0	23.0
≥ 65	11.1	56.5	32.5	12.4	56.4	31.2
Schooling*						
High	20.0	60.3	19.7	31.2	54.5	14.3
Low	14.5	58.6	26.9	20.4	57.0	22.6
Per capita income (mean and standard deviation)***	3,646.12 ± 2,625.29	2,625.29 ± 2,991.99	3,567.55 ± 3,050.03	4,229.28 ± 3,235.25	3,984.41 ± 3,169.99	3,563.11 ± 3,142.55
Self-reported race/skin colour*						
Non-white	34.1	12.2	53.7	22.2	56.0	21.8
White	21.1	60.5	21.1	30.1	55.3	14.6
Marital status***						
Not partnered	19.0	60.2	20.8	24.5	55.3	20.2
Partnered	17.0	59.4	23.6	27.9	55.9	16.2
Employment situation*						
Active	18.5	60.0	21.5	29.1	55.4	15.5
Retired	10.3	56.6	33.1	15.9	56.7	27.4
Tobacco use*						
Never smoked	20.1	60.5	19.3	28.3	55.0	16.6
Former smoker	13.8	57.5	28.7	22.6	56.0	21.4
Current smoker	15.9	61.3	22.8	23.4	58.1	18.4
**Alcohol abuse**						
No	17.8	58.9	23.3	26.6	55.4	18.1
Yes	13.9	64.1	22.0	20.1	64.4	15.5
Ate fruit daily***						
No	17.3	61.0	21.7	29.0	55.9	15.1
Yes	17.4	57.9	24.7	24.9	55.5	19.6
Ate vegetables daily***						
No	16.8	60.8	22.4	27.3	54.8	17.9
Yes	18.0	58.0	24.0	25.7	56.2	18.1
Anthropometric status**						
Non-obese	20.5	61.0	18.4	31.9	55.5	12.6
Obese	7.0	54.5	38.4	13.0	55.9	31.1
Hypertriglyceridemia*						
No	21.1	60.5	18.4	30.1	55.6	14.3
Yes	11.1	57.9	31.0	12.0	55.6	32.4
Arterial hypertension*						
No	23.6	63.0	13.4	33.5	56.1	10.3
Yes	9.6	55.1	35.3	14.4	54.8	30.8

*p<0.05 for both sexes; **p<0.05 for men; ***p<0.05 for women.

**Table 2:** – Crude and adjusted odds ratios (ORs) and respective 95% confidence intervals (95%CI) of the association between physical activity intensity trajectories and diabetes classification in men and women; Longitudinal Study of Adult Health (ELSA-Brasil), 2008-2019

Classification	Physical activity intensity trajectory	Men		Women	
		Crude OR (95%CI)	Adjusted OR *(95%CI)	Crude OR (95%CI)	Adjusted OR **(95%CI)
Prediabetes/ Normal	Strong	0.99 (0.68; 1.44)	1.18 (0.81; 1.74)	0.71 (0.47; 1.07)	0.83 (0.54; 1.26)
	Weak	1.24 (1.00; 1.54)	1.36 (1.09; 1.69)	1.17 (0.97; 1.41)	1.20 (0.99; 1.45)
Diabetes/ Prediabetes	Strong	0.53 (0.34; 0.83)	0.63 (0.40; 0.98)	0.28 (0.12; 0.68)	0.33 (0.14; 0.79)
	Weak	1.04 (0.85; 1.27)	1.10 (0.89; 1.35)	1.03 (0.83; 1.27)	1.03 (0.82; 1.28)

* adjusted for age group, schooling and self-reported race/color. **adjusted for schooling, self-reported race/color, employment situation and per capita income.
